# Effect of vNOTES hysterectomy with uterosacral ligament suspension on overactive bladder symptoms in patients with pelvic organ prolapse: a retrospective cohort study

**DOI:** 10.55730/1300-0144.6361

**Published:** 2026-07-11

**Authors:** Gonca TÜRKER ERGÜN, Mahmut KUNTAY KOKANALI, Duygu TUĞRUL ERSAK, Melike DOĞANAY, Özlem MORALOĞLU TEKİN

**Affiliations:** Department of Obstetrics and Gynecology, Ankara Bilkent City Hospital, Ankara, Turkiye

**Keywords:** Pelvic organ prolapse, vNOTES, uterosacral ligament suspension, overactive bladder

## Abstract

**Background/aim:**

To evaluate the anatomical and functional outcomes following vaginal natural orifice transluminal endoscopic surgery (vNOTES) hysterectomy combined with bilateral uterosacral ligament suspension in patients with apical prolapse and concomitant overactive bladder symptoms.

**Materials and methods:**

This retrospective cohort study was conducted at a single tertiary referral center. Women with symptomatic apical prolapse (stage ≥ II) and concomitant overactive bladder symptoms were included. All patients underwent vNOTES hysterectomy with bilateral uterosacral ligament suspension. Anatomical outcomes were assessed using the Pelvic Organ Prolapse Quantification (POP-Q) system, whereas functional outcomes were evaluated using validated questionnaires, including the Pelvic Floor Distress Inventory-20 (PFDI-20), Overactive Bladder Questionnaire-V8 (OAB-V8), and Overactive Bladder Symptom Score (OABSS).

**Results:**

A total of 25 patients were included in the study. Significant improvements were observed across all POP-Q system parameters at the 3-month postoperative follow-up. Furthermore, assessments of pelvic organ prolapse and overactive bladder symptoms using the PFDI-20, OAB-V8, and OABSS revealed statistically significant postoperative improvements compared with baseline. Overall, among all POP-Q measurements, only the change in point C was significantly associated with improvements in overactive bladder symptom scores measured by the OAB-V8 and OABSS (r = 0.642, p = 0.001; r = 0.589, p = 0.002, respectively).

**Conclusion:**

The vNOTES approach to bilateral uterosacral ligament suspension appears to be an effective minimally invasive technique that not only restores apical anatomical support but also substantially alleviates overactive bladder symptoms. Improvement in apical support may be a key determinant of functional recovery.

## 1. Introduction

Pelvic organ prolapse (POP) is a highly prevalent pelvic floor disorder that affects millions of women worldwide and represents a substantial clinical and socioeconomic burden. Its incidence increases with age, parity, and connective tissue weakening. Epidemiological data suggest that nearly half of parous women exhibit some degree of pelvic support defect [1,2]. Beyond its anatomical manifestations, POP is frequently accompanied by lower urinary tract symptoms, among which overactive bladder (OAB) is one of the most distressing and quality-of-life–limiting conditions [2].

The coexistence of POP and OAB is well established; however, the relationship between these entities remains controversial. Population-based and imaging studies have demonstrated that women with POP have a significantly higher prevalence of OAB symptoms, with reported prevalence rates ranging from 22% to over 50% [3]. In this context, the role of the apical compartment—and particularly the uterosacral ligaments—has gained considerable attention. Contemporary anatomical and functional models, including the updated integral theory, propose that laxity or failure of the apical support structures may lead to abnormal activation of bladder stretch receptors, thereby triggering urgency and detrusor overactivity [4].

Surgical management of POP has traditionally focused on restoring anatomical integrity; however, there is growing recognition that functional outcomes—particularly urinary symptoms—are equally critical determinants of surgical success. Uterosacral ligament suspension (USLS) remains one of the most widely used techniques for apical repair and has demonstrated favorable anatomical outcomes across multiple studies and metaanalyses [5,6]. Nevertheless, the extent to which apical reconstruction directly contributes to the resolution of OAB symptoms remains unclear, and the existing evidence is heterogeneous.

More recently, vaginal natural orifice transluminal endoscopic surgery (vNOTES) has emerged as an innovative minimally invasive approach in pelvic reconstructive surgery [7]. The vNOTES-USLS procedure, used for the surgical management of apical prolapse, has demonstrated favorable anatomical outcomes with a low complication profile and is being increasingly adopted in clinical practice [8]. However, there are limited data on the effects of this procedure on pelvic floor dysfunction associated with pelvic organ prolapse.

Given these gaps, a critical question remains unresolved: does restoration of apical support—particularly via advanced minimally invasive approaches such as vNOTES-USLS—translate into meaningful improvement in bladder function? Therefore, the present study aimed to evaluate the anatomical and functional outcomes following vNOTES hysterectomy combined with USLS in patients with apical prolapse and preoperative OAB symptoms. Additionally, this study sought to explore the relationship between restoration of apical support and changes in OAB symptom severity, based on the hypothesis that improved apical support may represent a key determinant of postoperative functional recovery.

## 2. Materials and methods

This retrospective cohort study included women who underwent vNOTES hysterectomy with concomitant USLS for stage ≥ II apical prolapse causing vaginal bulging, pressure, and discomfort at the Gynecology and Obstetrics Clinic of Ankara Bilkent City Hospital over a two year period and who had preoperative OAB symptoms. Inclusion criteria included age >40 years, no prior treatment for POP or urinary incontinence (surgical and conservative), and no history of pelvic surgery. Although all women were informed about uterine-preserving procedures prior to surgery, all opted for hysterectomy. Patients with missing baseline or follow-up data and those with stress urinary incontinence were excluded. Ethical approval was obtained from the Ankara Bilkent City Hospital Ethics Committee no. 2 (24–638). Informed consent was obtained from the participants.

All study data were obtained from hospital records. The sample size was determined by the number of consecutive eligible patients who met the inclusion criteria during the study period. Demographic and clinical characteristics included age, body mass index (BMI), parity, medical and surgical history, menopausal status, and lifestyle habits. Preoperative physical and gynecological examination findings and quality-of-life (QoL) questionnaire results related to patients’ symptoms were recorded. Operative data included the type of anesthesia, duration of surgery, concomitant procedures (salpingectomy ± oophorectomy, colporrhaphy, or other procedures), and intraoperative complications. Postoperative hospital stay, data from follow-up visits at 6 weeks and 3 months, and hospital readmissions during these periods were also analyzed. Examination findings, patients’ symptoms, repeated QoL questionnaire results, reasons for readmission, and complications were recorded. To ensure data consistency and accuracy, 10% of records were checked for errors after data entry was completed.

Preoperative evaluations, surgical procedures, and postoperative follow-up assessments were conducted by two surgeons, each with over 15 years of experience and specialized expertise in pelvic reconstructive surgery. The diagnosis of OAB was established based on clinical evaluation and patient-reported symptoms in accordance with the International Continence Society (ICS) definition. All women reported urinary frequency (≥8 voids per 24 h), nocturia (≥1 void per night), and urgency, with or without urinary incontinence, in their medical history [9]. These symptoms were objectively confirmed using bladder diaries. POP was evaluated using the Pelvic Organ Prolapse Quantification (POP-Q) system [10]. Postoperative recurrence was defined as the presence of stage ≥ II prolapse in any vaginal compartment according to the POP-Q classification.

Validated questionnaires, including the Pelvic Floor Distress Inventory-20 (PFDI-20) [11], Overactive Bladder Symptom Score (OABSS) [12], and Overactive Bladder Questionnaire-V8 (OAB-V8) [13], were used to assess preoperative and postoperative outcomes. The PFDI-20 was used to evaluate the impact of POP on QoL. This instrument consists of 20 items divided into three subscales: the Pelvic Organ Prolapse Distress Inventory-6 (POPDI-6), the Colorectal-Anal Distress Inventory-8 (CRADI-8), and the Urinary Distress Inventory-6 (UDI-6). Responses were initially recorded as “no” (0) or “yes”. If the response was “yes,” patients were asked to rate the degree of bother on a 4-point scale ranging from “not at all” (1) to “quite a bit” (4). The total PFDI-20 score ranges from 0 to 300, with each subscale ranging from 0 to 100. Higher scores indicate greater symptom severity and more severe pelvic floor dysfunction.

The severity of OAB symptoms was assessed using the OABSS, which consists of four items evaluating daytime voiding frequency, nocturia, urgency, and urge urinary incontinence. The total OABSS score ranges from 0 to 15, with higher scores reflecting increased symptom severity. Additionally, the impact of OAB symptoms on QoL was assessed using the OAB-V8 questionnaire. This eight item instrument uses a 6-point Likert scale ranging from “none” (0) to “very much” (5), yielding total scores ranging from 0 to 40. Higher scores indicate greater symptom burden. The OABSS and OAB-V8 questionnaires were not used as diagnostic tools or for patient selection; rather, they were administered to assess symptom severity and postoperative changes.

All procedures were performed by two surgeons with extensive experience in vNOTES, using standardized surgical protocols. Following the administration of prophylactic antibiotics, patients were placed in the Trendelenburg lithotomy position under general anesthesia. Intraperitoneal pressure was maintained between 10–12 mmHg throughout the procedure. Initially, the previously described surgical technique was followed, and vNOTES hysterectomy was performed using a GelPOINT V-Path port (Applied Medical, Rancho Santa Margarita, CA, USA) [14]. Subsequently, bilateral ureters and uterosacral ligaments were identified. In cases where the ureter was located in close proximity to the ipsilateral uterosacral ligament, a peritoneal incision was made to achieve ureteral lateralization. The cranial one-third of the uterosacral ligament, representing its strongest portion, was grasped approximately 1 cm above the ischial spine. Absorbable polydioxanone sutures (2–0, Ethicon LLC, Raritan, NJ, USA) were placed bilaterally. After removal of the port, the sutures were retrieved through the vaginal canal. Each suture from the uterosacral ligaments was anchored to the ipsilateral anterior and posterior vaginal cuff mucosa. Anterior colporrhaphy was performed when indicated. Following closure of the vaginal cuff with 1–0 polyglactin sutures, the bilateral uterosacral ligament sutures were tied to suspend the vaginal apex. Cystoscopy was then performed to confirm ureteral patency by visualization of ureteral efflux. Finally, posterior colporrhaphy was performed when indicated.

All statistical analyses were performed using IBM SPSS Statistics software (version 22.0; IBM Corp., Armonk, NY, USA). The distribution of continuous variables was evaluated using the Shapiro–Wilk test. Normally distributed data were presented as mean ± standard deviation, whereas nonnormally distributed data were presented as median (range). Categorical variables were summarized as n (%). For comparisons between preoperative and postoperative measurements, the paired-samples t-test was used for normally distributed variables. Categorical variables were compared using the chi-square test or Fisher’s exact test, as appropriate. Associations between changes in POP-Q points and changes in OABSS and OAB-V8 scores were assessed using Spearman’s correlation coefficient. A p < 0.05 was considered statistically significant.

## 3. Results

A total of 25 patients were included in the study. The median age was 52 years (range, 45–72), the median parity was 3 (range, 1–5), and the mean BMI was 30.2 kg/m^2^ (range, 27.8–33.4). Among the study population, 56.0% of patients were postmenopausal, 48.0% had a systemic disease, and 16.0% were smokers. The median operative time was 110 min (range, 85–145), and the median length of hospital stay was 2 days (range, 1–3). The most commonly performed concomitant procedure was bilateral salpingo-oophorectomy (80.0%) (Table 1).

Regarding apical POP stage, preoperatively, 76.0% of patients were classified as stage II and 24.0% as stage III–IV. At the 6-week postoperative follow-up, 92.0% of patients had improved to stage 0–1, and this rate was maintained at the 3-month postoperative follow-up (88.0%). Recurrence was observed in two patients at the 6-week follow-up and in three patients at the 3-month follow-up. All of these recurrences were in the anterior compartment; however, no patients required reoperation during the follow-up period (Table 1). Although no intraoperative complications were identified, postoperative urinary tract infections occurred in five patients, and vaginal cuff hematomas occurred in four patients; all resolved spontaneously. Following anterior compartment prolapse repair, one patient required 1 day of bladder training because of postoperative voiding difficulty and was discharged without complications on the 3rd postoperative day.

Comparison of POP-Q measurements at the 3-month postoperative follow-up demonstrated statistically significant improvements in all parameters. There were marked improvements in points Aa, Ba, and C (all p < 0.001). Posterior compartment measurements, including points Ap and Bp, also showed significant improvements (p = 0.001 and p < 0.001, respectively). In addition, total vaginal length (TVL) increased significantly postoperatively (p < 0.001) (Table 2).

The questionnaire scores showed significant improvements at the 3-month postoperative follow-up. The total PFDI-20 score and all its subscales (POPDI-6, CRADI-8, and UDI-6) decreased significantly after surgery (all p < 0.001). Similarly, all components of the OABSS demonstrated significant reductions, including daytime and nighttime urinary frequency, urgency, and urge urinary incontinence (all p ≤ 0.004). The total OABSS score decreased from 7.64 ± 1.44 to 4.12 ± 1.05 (p < 0.001). Furthermore, the total OAB-V8 score decreased significantly from 21.96 ± 1.90 to 11.12 ± 2.32 (p < 0.001) (Table 3).

Correlation analyses between changes in POP-Q measurements and questionnaire scores indicated a statistically significant positive correlation between Δpoint C and both ΔOABSS score (r = 0.589, p = 0.002) and ΔOAB-V8 score (r = 0.642, p = 0.001). No statistically significant correlations were observed between the other POP-Q parameters (Δpoint Aa, Δpoint Ba, Δpoint Ap, Δpoint Bp, and ΔTVL) and ΔOABSS or ΔOAB-V8 scores (p > 0.05 for all) (Table 4).

## 4. Discussion

This study yielded several important findings. The results demonstrated that the vNOTES-USLS procedure, performed for the surgical management of apical prolapse, not only provided robust anatomical correction but also was associated with clinically significant improvement in concomitant OAB symptoms. More importantly, the improvement in bladder function appears to be more closely associated with the restoration of apical support rather than correction of the anterior compartment. In this context, the vNOTES-USLS procedure may be considered a potentially beneficial approach for the anatomical and functional management of bladder dysfunction secondary to apical prolapse, as it provides effective apical support. These findings help address an important gap in the existing literature and contribute to the ongoing debate regarding the pathophysiological relationship between POP and OAB and their management.

While previous studies have reported improvements in urinary symptoms following prolapse surgery [15,16], the underlying mechanisms remain incompletely understood, and the reported outcomes are often inconsistent [17]. A major limitation of earlier studies is their predominant focus on global anatomical success without evaluating the relative contributions of individual pelvic compartments [15–17]. In contrast, the present study specifically evaluated the relationship between compartment-specific anatomical changes and functional outcomes, thereby providing additional mechanistic insight.

Consistent with recent literature [8,18], we found that the vNOTES-USLS procedure performed after hysterectomy provided effective and reliable short-term anatomical outcomes in the surgical treatment of apical prolapse. Importantly, this anatomical success was accompanied by functional improvement, as reflected by significant reductions in OAB symptom severity. Notably, improvement in apical support (point C) was significantly associated with symptom relief, whereas no similar relationship was observed for the anterior compartment parameters (points Aa and Ba). This finding is consistent with previous studies suggesting a stronger relationship between apical prolapse and urinary symptoms compared to anterior compartment defects [19,20]. From a mechanistic perspective, this observation may be explained by the integral theory, which proposes that laxity of the uterosacral ligaments leads to altered tension within the pelvic support system, resulting in inappropriate activation of stretch receptors in the bladder base and trigone [21–23]. Restoration of apical support may therefore normalize these biomechanical forces and reduce involuntary detrusor activity.

Furthermore, emerging imaging and functional studies have highlighted the role of apical descent and pelvic floor muscle dysfunction in the pathophysiology of urgency-related symptoms [5,24,25]. This study extends the current evidence by demonstrating that targeted correction of apical support was associated with measurable improvement in OAB symptoms, supporting the concept that apical compartment integrity may play a central role in lower urinary tract function.

The surgical approach used in this study—vNOTES—may further enhance these effects. Compared with conventional vaginal or laparoscopic techniques, vNOTES offers superior visualization of deep pelvic structures, enabling precise identification and strong, symmetrical suspension of the uterosacral ligaments at an optimal anatomical level [8,18,26]. This technical advantage may contribute to more effective restoration of apical support, as reflected by the significant improvement in point C measurements observed in this study. Furthermore, vNOTES facilitates suspension using the cranial, stronger portion of the uterosacral ligament, which may contribute to reducing ligament laxity and improving OAB symptoms, as proposed by the integral theory. In addition, the minimally invasive nature of vNOTES, characterized by reduced tissue dissection and improved preservation of neurovascular structures, may facilitate faster recovery of bladder function [8,27]. The absence of significant intraoperative and postoperative complications in this study suggests that vNOTES is a reliable and minimally invasive surgical approach. However, current evidence remains insufficient to recommend its routine use over conventional laparoscopic or vaginal approaches. Individualized surgical planning based on prolapse characteristics, patient symptoms, and surgeon expertise remains essential. Prospective comparative studies are needed to determine whether the observed functional benefits are attributable to the vNOTES approach itself or to restoration of apical support.

Anterior compartment prolapse recurrence was observed in three patients during follow-up. All recurrences were identified anatomically based on the POP-Q examination, and none required reoperation during the study period. Furthermore, only a small proportion of patients underwent concomitant anterior and/or posterior colporrhaphy. While the primary objective of the present study was to evaluate the relationship between apical support restoration and overactive bladder symptoms, the potential contribution of these concomitant compartment-specific repairs to postoperative symptom improvement cannot be completely excluded and should be considered when interpreting our findings.

Despite these strengths, several limitations should be acknowledged. The retrospective design introduces the possibility of selection bias and limits causal inference. The absence of a control group limits direct comparisons of surgical outcomes. The relatively small sample size may reduce statistical power and limit the generalizability of the findings. OAB symptoms were assessed using patient-reported questionnaires, which, although validated, remain inherently subjective. Additionally, the follow-up period was relatively short, precluding the assessment of the long-term durability of both anatomical and functional outcomes. Future studies should include prospective, randomized controlled trials comparing vNOTES-USLS with other surgical approaches to better establish its comparative efficacy and safety. In addition, incorporating urodynamic studies would provide an objective evaluation of postoperative bladder function and complement patient-reported symptom assessments. The present study was limited by a 3-month follow-up period, which may not fully capture the long-term durability of the anatomical and functional outcomes; therefore, studies with follow-up periods exceeding 12 months are warranted.

Nevertheless, this study has several notable strengths. It provides a comprehensive evaluation of anatomical and functional outcomes and incorporates a compartment-specific analysis. Importantly, it focuses on a minimally invasive surgical approach, vNOTES, that is gaining increasing clinical relevance.

Taken together, these findings have important clinical implications. They suggest that surgical planning for POP should aim not only to achieve anatomical correction but also to consider the functional impact of compartment-specific repair. In particular, restoration of apical support may be critical in patients presenting with concomitant OAB symptoms. The vNOTES-USLS procedure following hysterectomy is a minimally invasive surgical approach that provides enhanced visualization, facilitates suspension at an appropriate anatomical level, and may contribute to both anatomical and functional improvement. This perspective may help refine patient selection, optimize surgical strategies, and improve overall treatment outcomes.

In conclusion, apical prolapse and OAB are closely associated pelvic floor disorders. In patients with apical prolapse and concomitant OAB symptoms, vNOTES-USLS appears to be an effective and reliable surgical approach for correcting anatomical defects and improving both POP and OAB symptoms. Future prospective, randomized studies with larger sample sizes and longer follow-up are warranted to validate these findings and further elucidate the mechanistic relationship between apical support and bladder function.

## Figures and Tables

**Table 1:** Demographic, operative, and follow-up characteristics of the study population.

N= 25	Preoperative baseline characteristics		

Age (years)	52 (45–72)		

Parity	3 (1–5)		

BMI (kg/m^2^)	30.2 (27.8–33.4)		

Postmenopausal status	14 (56.0)		

Systemic comorbidity	12 (48.0)		

Current smoker	4 (16.0)		

Operative time (min)	110 (85–145)		

Concomitant procedures			
Bilateral salpingo-oophorectomy	20 (80.0)		
Bilateral salpingectomy	2 (8.0)		
Anterior colporrhaphy	2 (8.0)		
Posterior colporrhaphy	6 (24.0)		
Hospital stay (days)	2 (1–3)		

	Preoperative baseline characteristic	6-week postoperative follow-up	3-month postoperative follow-up

Apical POP-Q stage (apical compartment)			

Stage 0-I	0 (0.0)	23 (92.0)	22 (88.0)

Stage II	19 (76.0)	2 (8.0)	3 (12.0)

Stage III- IV	6 (24.0)	0 (0.0)	0 (0.0)

Recurrence*	---	2 (8.0)	3 (12.0)

Reoperation	---	0 (0.0)	0 (0.0)

Values are presented as mean ± standard deviation, median (range), or n (%).

BMI: body mass index.

* All recurrences occurred in the anterior compartment.

**Table 2:** Preoperative and 3-month postoperative POP-Q measurements (N = 25).

	Preoperative	Postoperative	p
Aa	−0.40±1.41	−2.60±0.50	<0.001
Ba	0.32±2.08	−2.36±0.57	<0.001
C	0.40±2.92	−6.32±0.80	<0.001
Ap	−1.28±1.07	−2.12±0.78	0.001
Bp	−0.56±1.78	−2.16±0.69	<0.001
TVL	6.80±0.82	7.56±0.71	<0.001

POP-Q: Pelvic Organ Prolapse Quantification; TVL: total vaginal length.

Values are presented as mean ± standard deviation.

p < 0.05 was considered statistically significant.

**Table 3:** Preoperative and 3-month postoperative questionnaire results.

		Preoperative	Postoperative	p
PFDI-20 Scores	POPDI-6 score	9.9 ± 3.5	0.9 ± 1.9	<0.001
	CRADI-8 score	2.5 ± 3.0	0.7 ± 2.1	<0.001
	UDI-6 score	7.5 ± 4.4	1.6 ± 2.8	<0.001
	Total score	19.9 ± 6.7	3.2 ± 5.4	<0.001
OABSS Scores	Daytime voiding score	1.00±0.29	0.24±0.44	<0.001
	Nighttime voiding score	1.76±0.52	0.64±0.57	<0.001
	Urgency score	2.84±0.55	1.72±0.46	<0.001
	Urge urinary incontinence score	2.04±0.73	1.52±0.51	0.004
	Total score	7.64±1.44	4.12±1.05	<0.001
OAB-V8 Scores	Total score	21.96±1.90	11.12±2.32	<0.001

PFDI-20: Pelvic Floor Distress Inventory-20; POPDI-6: Pelvic Organ Prolapse Distress Inventory-6; CRADI-8: Colorectal-Anal Distress Inventory-8; UDI-6: Urinary Distress Inventory-6; OABSS: Overactive Bladder Symptom Score; OAB-V8: Overactive Bladder Questionnaire-V8.

Values are presented as mean ± standard deviation.

p < 0.05 was considered statistically significant.

**Table 4:** Correlations between changes in POP-Q measurements and questionnaire scores.

	ΔOABSS Score		ΔOAB-V8 Score	
	r	p	r	p
ΔAa	0.214	0.305	0.384	0.388
ΔBa	0.216	0.300	0.398	0.342
ΔC	0.589	0.002	0.642	0.001
ΔAp	0.133	0.527	0.425	0.252
ΔBp	0.073	0.730	0.320	0.404
ΔTVL	0.210	0.313	0.326	0.421

Δ: postoperative–preoperative value; OABSS: Overactive Bladder Symptom Score; OAB-V8: Overactive Bladder Questionnaire-V8; TVL: total vaginal length.

r: Spearman’s correlation coefficient.

p < 0.05 was considered statistically significant.

## Data Availability

The datasets generated and/or analyzed during the current study are available from the corresponding author upon reasonable request.
